# 35 Years of Joyner’s Endurance Performance Model: Assessing the Contribution of Physiological Determinants of Performance Proxies in 888 Individuals from Recreational to World Class

**DOI:** 10.1007/s40279-026-02439-y

**Published:** 2026-05-03

**Authors:** Loïs Mougin, Stephen J. Bailey, Andrew M. Jones, Michael J. Joyner, Stephen A. Mears, Rhona Pearce, Michele Zanini

**Affiliations:** 1 School of Sport, Exercise and Health Sciences, Loughborough University, Clyde Williams Building, Epinal Way, Loughborough, Leicestershire LE11 3TU UK; 2 Department of Public Health and Sport Sciences, University of Exeter, Exeter, UK; 3 Department of Anesthesiology and Perioperative Medicine, Mayo Clinic, Rochester, MN USA; 4 Loughborough Sport, Loughborough University, Loughborough, UK; 5 School of Education, Childhood, Youth and Sport, The Open University, Milton Keynes, UK

## Abstract

**Background:**

Endurance performance is predicted by maximal oxygen uptake, its fractional utilisation at lactate threshold (FU_LT_) and exercise economy. These variables are used to estimate speed or power at lactate threshold (LT) and lactate turnpoint (LTP), which serve as performance proxies.

**Objective:**

This study examined the relationships between these variables in a large cohort of runners and cyclists and quantified their relative contributions to performance prediction.

**Methods:**

495 runners (105 females) and 393 cyclists (42 females) completed incremental exercise tests to determine maximal oxygen uptake (running [R]: 56 mL/kg/min, 3.94 L/min; cycling [C]: 52 mL/kg/min, 3.99 L/min), economy (R: 220 mL/kg/km; C: 14.7 mL/min/W), FU_LT_ (R: 78%; C: 70%), FU_LTP_ (R: 88%; C: 84%), and speed or power at LT (R: 12.0 km/h; C: 190 W) and LTP (R: 13.9 km/h; C: 240 W). Single and multiple linear regression models were used to examine the relationship and relative contribution of physiological determinants to performance proxies.

**Results:**

Speed or power at LT and LTP correlated strongly and positively with maximal oxygen uptake (*R*^2^ = 0.65–0.77; *P* < 0.001), and inversely with economy (*R*^2^ = 0.24–0.26; *P* < 0.001). In contrast, trivial relationships were observed with FU_LT_ (*R*^2^ ≤ 0.04; *P* = 0.01–0.05) or FU_LTP_ (*R*^2^ ≤ 0.01; *P* = 0.01–0.09). Regression models estimating LT and LTP from physiological determinants showed very strong agreement with measured performance proxies (*R*^2^ = 0.94–0.99; *P* < 0.001), indicating consistency in their relative contribution to performance proxies. Maximal oxygen uptake contributed most to performance proxies (65–76%) followed by running economy (20–24%), with marginal contributions from FU_LT_ or FU_LTP_ (4–11%).

**Conclusions:**

These results indicate that maximal oxygen uptake and economy collectively predict ~ 95% of speed or power at LT and LTP, and by extension performance, whilst the contribution of FU_LT_ or FU_LTP_ is limited in populations with heterogeneous characteristics.

**Supplementary Information:**

The online version contains supplementary material available at 10.1007/s40279-026-02439-y.

## Key Points

**Table:** 

Maximal oxygen uptake is the dominant determinant of endurance performance in both running and cycling, accounting for the majority (65–76%) of inter-individual differences in speed or power at lactate threshold and lactate turnpoint, used here as performance proxies.	
Exercise economy also substantially contributes to endurance performance, with running and cycling economy accounting for 20–25% of variance in performance proxies.	
Fractional utilisation of maximal oxygen uptake contributes marginally to performance differences in heterogeneous cohorts, highlighting its limited discriminative value for predicting performance among athletes of varying abilities, although it remains relevant for profiling elite athletes.	
Regression models integrating maximal oxygen uptake, exercise economy (running economy/cycling economy), and fractional utilisation (at lactate threshold/lactate turnpoint) explained almost all the variance in speed or power at lactate threshold and lactate turnpoint, indicating a remarkably high consistency in their relative contribution to performance proxies across participants of heterogeneous characteristics.	

## Introduction

In 1991, Joyner proposed a now-classic model describing endurance performance as a product of three key physiological determinants: (i) maximal oxygen uptake (${\dot{\mathrm{V}}}{\mathrm{O}}_2 {\mathrm{max}}$); (ii) the fractional utilisation of ${\dot{\mathrm{V}}}{\mathrm{O}}_2 {\mathrm{max}}$ at lactate threshold (FU_LT_) and (iii) exercise economy (or efficiency) [1]. This framework, originally developed to predict marathon performance, has since become a foundational model in physiology applied to endurance sports, particularly in events characterised by relatively steady pacing strategies. According to the model, ${\dot{\mathrm{V}}}{\mathrm{O}}_2 {\mathrm{max}}$ defines the upper limit for aerobic metabolism, FU_LT_ or fractional utilisation at lactate turnpoint (FU_LTP_) represents the sustainable fraction of ${\dot{\mathrm{V}}}{\mathrm{O}}_2 {\mathrm{max}}$ and demarcates exercise intensity domains [2], and exercise economy determines the oxygen cost of producing a given speed or power output. In Joyner’s model, these three physiological factors are used to estimate the speed at lactate threshold (LT), considered a marathon performance proxy given its large positive association with marathon speed [3, 4] as well as cycling performance (e.g. ~ 30-min time trial) [5]. Similarly, the speed or power at lactate turnpoint (LTP) can be calculated from ${\dot{\mathrm{V}}}{\mathrm{O}}_2 {\mathrm{max}}$, exercise economy and FU_LTP_, and can be considered a performance proxy for events lasting 20–60 min [6, 7].

Over the past three decades, Joyner’s conceptual framework has not only influenced scientific understanding and approaches to enhance endurance performance but has demonstrated excellent predictive power, having been used to accurately predict world-record performances [3, 4]. Later iterations of the model integrated the putative physiological processes underpinning each of the performance determinants, including cardiovascular, metabolic and (neuro)muscular factors [8]. Although Joyner’s model has been recently extended given that these determinants may change during prolonged exercise [9, 10], with durability/physiological resilience suggested to be a potential fourth factor determining endurance performance [11–13], the classical physiological determinants remain fundamental for understanding endurance performance.

A high ${\dot{\mathrm{V}}}{\mathrm{O}}_2 {\mathrm{max}}$ and excellent economy are characteristic features of high-performing endurance athletes and clearly distinguish between higher and lower calibre athletes [14–17]. Conversely, the extent to which FU_LT_ and FU_LTP_ can discriminate athletes of heterogeneous performance levels has been challenged [18–20]. Importantly, elite endurance athletes may display similar performance despite substantial inter-individual variability in physiological determinants, suggesting that diverse physiological make-ups may contribute to performance outcomes [3, 21]. Despite the enduring influence of Joyner’s model, most supporting studies have been limited by small sample sizes (often < 20 participants) and homogeneous male cohorts [1, 5, 21]. These methodological limitations have impeded our understanding of how each physiological determinant contributes to performance, and whether differences exist across performance levels, exercise modality and biological sex.

Sex differences have been reported for running economy (RE) [14], ${\dot{\mathrm{V}}}{\mathrm{O}}_2 {\mathrm{max}}$ [22, 23], critical power [24, 25], muscle size and fibre-type composition, cardiorespiratory anatomy and haemoglobin mass [26, 27], as well as endurance performance; consequently, exploring the proportional contribution of physiological determinants in a sex-specific manner may highlight differences with potential implications for training and racing strategies. Similarly, physiological determinants have been shown to differ depending on exercise modalities. For example, cycling generally elicits lower ${\dot{\mathrm{V}}}{\mathrm{O}}_2 {\mathrm{max}}$ values than running, owing to reduced active muscle mass and mechanical constraints [28]. These modality-specific differences suggest that the relative importance of physiological determinants may differ across exercise modalities or sports. Addressing these limitations, therefore, has the potential to improve the translational potential of the Joyner model.

As speed or power at LTP is closely associated with endurance performance, attempts have been made to estimate these parameters from training and racing data [29], with assessments of critical power/speed having been shown to broadly correspond to LTP [30–32]. Although LT also reflects endurance performance, its estimation from field data has not been explored and currently still requires laboratory testing. Exploring the ratio between LT and LTP in a large population of athletes may, therefore, provide an indirect method for estimating LT. Furthermore, LT/LTP ratios may depend on performance levels, biological sex or exercise modality, and investigating these across different groups and exercise modalities could be useful for field-based performance monitoring.

Although the determinants of endurance performance are well established [1], no large-scale study has yet examined their relative contribution to performance variation for running and cycling in a large cohort of athletes, across sexes and performance levels. Therefore, the aim of this study was to explore the relationships between endurance-related physiological determinants (${\dot{\mathrm{V}}}{\mathrm{O}}_2 {\mathrm{max}}$, exercise economy, and FU_LT_ and FU_LTP_) with speed or power at LT and LTP as proxies of performance, in a large cohort of runners and cyclists from recreational to elite level. We also aimed to assess the relative contribution to performance variation of these physiological determinants on performance proxies, by examining the relationship between LT and LTP, and to analyse differences between sexes, exercise modalities and performance levels.

## Methods

Retrospective data from 888 participants were used in this study and included individuals from Tier 1 to Tier 5 according to the Participant Classification Framework of McKay et al. [33]. The data set included the 495 individuals (105 female) who completed an incremental running test and 393 individuals (42 female) who completed an incremental cycling test. All participants volunteered for laboratory-based exercise assessments conducted for physiological profiling, during which they provided verbal and written informed consent. As part of the consent process, participants were informed that their anonymised data may be used for future secondary analyses. The original data collection protocol received ethical approval from the Loughborough University Ethics Review Sub-Committee (Projects ID: G13-P1; 2020–1944-1940). Some of these data have been published previously for different outcomes [34, 35].

Participants performed incremental exercise tests to determine speed or power at LT and LTP, FU_LT_, FU_LTP_, exercise economy (RE or cycling economy [CE]), and ${\dot{\mathrm{V}}}{\mathrm{O}}_2 {\mathrm{max}}$. Each participant reported to the laboratory for a single visit. Participants were asked to avoid strenuous exercise in the 24 h before testing and to refrain from any physical activities on the day of the test. They were also advised to consume their usual pre-race meal and beverages and to avoid large meals within 3 h of testing.

### Running Test

The running incremental exercise test was performed on a treadmill (Mercury; h/p/cosmos, Nussdorf, Germany). The test comprised 4-min stages, interspersed by 30 s of rest for earlobe capillary blood sampling, until a respiratory exchange ratio > 1.0 was reached. Pulmonary gas exchange and ventilation were measured continuously throughout the test. Starting speed was 5.5–12.0 km/h and speed increments were 0.5–1.0 km/h, determined based on the participants’ training background. The gradient was fixed to 1% for the submaximal stages [36]. Following 15–20 min of rest after completion of the initial incremental test, another incremental test was performed to task failure to assess ${\dot{\mathrm{V}}}{\mathrm{O}}_2 {\mathrm{max}}$. The starting speed was set 2 km/h lower than the final stage of the submaximal test, with an initial incline of 1% [36], which increased thereafter by 1% every minute until task failure, aiming for a test duration of 8–12 min. The highest 1-min rolling average ${\dot{\mathrm{V}}}{\mathrm{O}}_2$ during the test was used to estimate ${\dot{\mathrm{V}}}{\mathrm{O}}_2 {\mathrm{max}}$, to reduce breath-by-breath variability.

### Cycling Test

The cycling incremental exercise test was performed either on a turbo trainer (Kickr Smart Trainer; Wahoo Fitness, Atlanta, GA, USA) using the participant’s own bicycle or on an SRM cycle ergometer (Schoberer Rad Messtechnik, Jülich, Germany), with similar power output accuracy demonstrated between the two devices [37]. The test comprised 4-min stages until a respiratory exchange ratio > 1.0 was reached. Starting power was 40–220 W, and the power increment between stages was 10–30 W, based on the participants’ training background. A capillary blood sample was collected from the earlobe in the last 30 s of each stage without exercise interruptions, and pulmonary gas exchange was measured continuously throughout the test. Following 15–20 min of rest after the end of the incremental test, a ramp test to task failure was performed to determine ${\dot{\mathrm{V}}}{\mathrm{O}}_2 {\mathrm{max}}$. The starting power (40–260 W) was determined based on training background, with an increase of 20 W/min for males or 15 W/min for females until task failure, aiming for a test duration of 8–12 min. The highest 1-min rolling average ${\dot{\mathrm{V}}}{\mathrm{O}}_2$ of the exercise was used to estimate ${\dot{\mathrm{V}}}{\mathrm{O}}_2 {\mathrm{max}}$.

### Gas Exchange and Blood Lactate Concentration

Throughout the test, participants wore a low-dead-space facemask and breathed through an impeller turbine assembly (Jaeger Triple V; Jaeger GmbH, Hoechberg, Germany) to measure O_2_ and CO_2_ concentrations in expired air via an open-circuit metabolic cart (Jaeger Vyntus CPX; Carefusion, San Diego, CA, USA). Inspired and expired gas volumes and concentrations were continuously measured on a breath-by-breath basis using paramagnetic (O_2_) and infrared (CO_2_) analysers (Jaeger Vyntus CPX) with a 75-ms typical rise time (T10–90), via a capillary line. These analysers were calibrated before each test using a known gas mixture (16% O_2_ and 5% CO_2_) and ambient air. The turbine volume transducer was calibrated using a 3-L syringe (Hans Rudolph Inc., Shawnee, KS, USA). The volume and concentration signals were time aligned, accounting for the transit delay in capillary gas and analyser rise time relative to the volume. Blood lactate concentration was analysed (Biosen C-Line Glucose and Lactate analyser; EKF Diagnostics, Cardiff, UK) from 20-μL earlobe capillary samples.

### Data Analysis

**Fig. 1:**
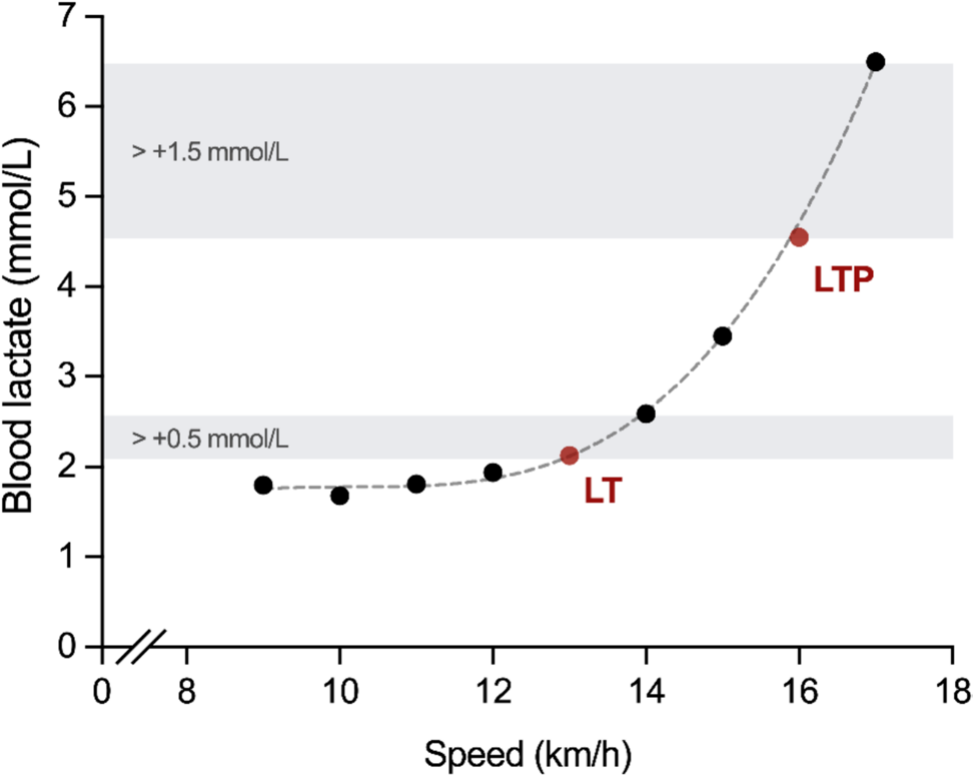
Typical blood lactate concentrations during an incremental running test, showing the analytical approaches used for identification of the lactate threshold (LT; increment > 0.5 mmol/L on subsequent stages) and lactate turnpoint (LTP; increment > 1.5 mmol/L on subsequent stages)

During the incremental test, LT was defined as the first rise in blood lactate concentration from baseline (+ 0.4–0.5 mmol/L), while LTP was defined as a rapid and sustained increase in blood lactate concentration (+ 1.5 mmol/L) between two subsequent stages [38] as described in Fig. 1. The mean ${\dot{\mathrm{V}}}{\mathrm{O}}_2$ collected during the final minute of the stage corresponding to LT was used to calculate the exercise economy. Running economy was expressed as mL/kg/km, while CE was expressed as mL/min/W. In running, ${\dot{\mathrm{V}}}{\mathrm{O}}_2 {\mathrm{max}}$ and exercise economy were expressed relative to body mass, whereas absolute values (i.e. not adjusted per body mass) were reported for cycling (cycling data relative to body mass are presented in the Electronic Supplementary Material [ESM]). This approach was used to account for the effect of body mass on the metabolic cost of running [39], while recognising that body mass exerts minimal influence on ${\dot{\mathrm{V}}}{\mathrm{O}}_2 {\mathrm{max}}$ during non-weight-bearing exercise such as cycling performed on a flat terrain [40]. The ratio between LT and LTP was calculated as speed or power at LT and LTP (% LTP at LT).

Participants were categorised into three performance tertiles based on their speed or power at LT. Within each sex, individuals were first ranked according to their speed or power at LT and then divided as evenly as possible into three groups. When a speed or power at LT value occurred at the boundary between tertiles, adjustments were made so that all athletes with the same value were placed in the same performance tertile. For running, males were divided as follows: ≤ 11.4 km/h (lowest tertile; *n* = 131), 11.5–13.0 km/h (middle tertile; *n* = 139) and ≥ 13.1 km/h (highest tertile; *n* = 120). Females were divided as follows: < 10.0 km/h (lowest tertile; *n* = 36), 10.2–11.6 km/h (middle tertile; *n* = 33) and ≥ 11.8 km/h (highest tertile; *n* = 36). For cycling, in males, lowest tertile corresponded to ≤ 179 W (*n* = 116), middle tertile to 180–209 W (*n* = 117) and Highest Tertile to ≥ 210 W (*n* = 118). In female cyclists, lowest tertile was set as ≤ 127 W (*n* = 13), middle tertile ranged between 130 and 160 W (*n* = 15) and highest tertile was defined as ≥ 162 W (*n* = 14).

### Statistical Analysis

Analyses were conducted in RStudio (Version 2023.03.0; RStudio, PBC, Boston, MA, USA). Multiple correlations were assessed using linear models, and the resulting *P*-values were corrected for multiple testing using the Holm–Bonferroni procedure. For the correlation matrix, *P*-values were not adjusted, to allow exploration of a broad set of relationships without restricting the analysis. Male and female characteristics were compared with unpaired t-tests. Linear mixed models were used to analyse differences between performance tertiles for physiological determinants and speed/power at LT/LTP and corrected for multiple comparisons with Bonferroni post-hoc.

To examine the relative contribution of physiological determinants to proxies of performance (as speed at LT and LTP for running and power at LT and LTP for cycling), a multiple linear regression model was constructed with speed or power at LT and LTP as the dependent variable and ${\dot{\mathrm{V}}}{\mathrm{O}}_2 {\mathrm{max}}$, RE/CE, and FU_LT_/FU_LTP_ at LT and LTP as independent variables. No collinearity between model predictors was found, which was assessed through variance inflation factor (< 1.1 for all predictors). A ten-fold cross-validation was also used to assess the model generalisability, with prediction errors averaged across folds and cross-validated R^2^ and root mean square error reported. Based on each variable’s coefficient in the multiple linear regression models, equations were developed to estimate performance proxies. The relative importance of each predictor was quantified using the Lindeman, Merenda, and Gold method implemented in the relaimpo package. This approach partitions the explained variance (*R*^*2*^) among predictors to determine their proportional contribution to overall model performance. Relative importance values were expressed as percentages of the total model *R*^*2*^.

## Results

### Participant Characteristics and Relationship Between Physiological Determinants and Performance Proxies

Participant and test characteristics are summarised in Table 1. In running, males exhibited better ${\dot{\mathrm{V}}}{\mathrm{O}}_2 {\mathrm{max}}$ (absolute and relative), speed at LT and LTP, and RE compared with females, while FU_LT_, FU_LTP_ and LT/LTP ratio were higher in females compared with males (all *P* < 0.05). For cycling, males exhibited greater absolute ${\dot{\mathrm{V}}}{\mathrm{O}}_2 {\mathrm{max}}$, power at LT and LTP than females, while females had higher FU_LT_ (all *P* < 0.05).

**Table 1:** Participant characteristics

Participant characteristics	Exercise modality					
	Running			Cycling		
	All	Males	Females	All	Males	Females
Sample size, *n*	495	390	105	393	351	42
Body mass, kg	71.3 (11.8) [39.6; 123.1]	74.2 (10.4) [46.0; 123.1]	60.2 (10.0) [39.6; 89.0]^a^	77.0 (11.6) [50.6; 116.8]	78.5 (10.9) [57.4; 116.8]	63.4 (8.9) [50.6; 85.2]^a^
Heart rate maximum, beats/min	182 (13) [147; 213]	181 (13) [147; 211]	185 (13) [155; 213]^a^	180 (13) [106; 208]	180 (13) [106; 208]	181 (10) [160; 197]
${\dot{\mathrm{V}}}{\mathrm{O}}_2 {\mathrm{max}}$, mL/min/kg	55.9 (9.0) [29.3; 80.7]	57.2 (8.6) [29.3; 80.7]	51.2 (8.7) [29.5; 69.1]^a^	52.4 (9.5) [26.5; 76.7]	52.6 (9.8) [27.4; 76.7]	49.9 (10.4) [26.5; 69.3]
${\dot{\mathrm{V}}}{\mathrm{O}}_2 {\mathrm{max}}$, L/min	3.94 (0.75) [1.70; 6.07]	4.19 (0.58) [2.35; 6.07]	2.98 (0.50) [1.70; 4.59]^a^	3.99 (0.66) [1.70; 6.20]	4.08 (0.62) [2.12; 6.20]	3.16 (0.62) [1.70; 4.36]^a^
RE, mL/kg/km – CE, mL/min/W	220 (18) [179; 292]	218 (18) [179; 289]	226 (15) [189; 269]^a^	14.7 (1.5) [10.2; 20.6]	14.6 (1.5) [10.2; 20.6]	14.8 (1.4) [12.4; 18.3]
Speed or power at LT, km/h or W	12.0 (2.2) [6.0; 18.5]	12.3 (2.2) [6.0; 18.5]	10.8 (1.8) [6.0; 14.4]^a^	190 (39) [85; 300]	195 (36) [92; 300]	155 (44) [85; 266]^a^
FU_LT_, %	78 (5) [59; 92]	77 (5) [60; 92]	79 (5) [67; 89]^a^	70 (6) [52; 90]	69 (6) [52; 90]	71 (6) [56; 83]
Heart rate at LT, beats/min	156 (14) [116; 196]	154 (13) [121; 187]	161 (15) [116; 196]^a^	142 (14) [88; 181]	142 (14) [88; 181]	148 (12) [124; 174]^a^
%HRmax at LT, %	86 (4) [68; 95]	85 (4) [73; 95]	87(4) [68; 95]^a^	80 (5) [64; 89]	80 (5) [64; 89]	83 (4) [71; 89]^a^
Speed, power at LTP, km/h – W	13.9 (2.4) [7.0; 20.5]	14.3 (2.4) [7.0; 20.5]	12.4 (2.2) [7.0; 17.0]^a^	240 (45) [102; 350]	246 (42) [127; 350]	192 (48) [102; 305]^a^
FU_LTP_, %	88 (6) [72; 99]	88 (4) [72; 99]	89 (4) [80; 97]^a^	84 (6) [58; 99]	84 (6) [59; 99]	85 (6) [73; 96]
Heart rate at LTP, beats/min	171 (13) [129; 205]	169 (13) [129; 202]	175 (14) [141; 205]^a^	160 (13) [98; 192]	160 (13) [98; 192]	165 (10) [139; 187]^a^
%HRmax at LTP, %	94 (3) [81; 100]	93 (3) [81; 100]	95 (3) [83; 100]^a^	89 (4) [77; 98]	89 (4) [77; 98]	92 (3) [85; 97]^a^
LT/LTP ratio, %	86 (3) [75; 94]	86 (3) [75; 94]	87 (3) [79; 94]^a^	79 (4) [67; 91]	79 (4) [67; 91]	79 (4) [67; 91]
*Submaximal incremental exercise*						
Start speed or power, km/h or W	9.8 (1.8) [5.5; 15.5]	10.0 (1.8) [5.5; 15.5]	9.0 (1.6) [6; 14]^a^	125 (29) [40; 180]	125 (27) [40; 180]	100 (27) [50; 180]^a^
Stage increment, km/h or W	0.8 (0.2) [0.5; 1.0]	0.8 (0.2) [0.5; 1.0]	0.8 (0.2) [0.5; 1.0]	25 (6) [10; 40]	25 (6) [10; 40]	17 (4) [10; 30]^a^

Mean (standard deviation) [minimum; maximum]

*CE* cycling economy, *FU*_*LT*_ and *FU*_*LTP*_ fractional utilisation of ${\dot{\mathrm{V}}}{\mathrm{O}}_2 {\mathrm{max}}$ at LT and LTP, respectively, *LT* lactate threshold, *LTP* lactate turnpoint, *RE* running economy, $\dot{{V}}{{O}}_2 {{max}}$ maximal oxygen uptake

^a^Different from males, *P* < 0.05

**Fig. 2:**
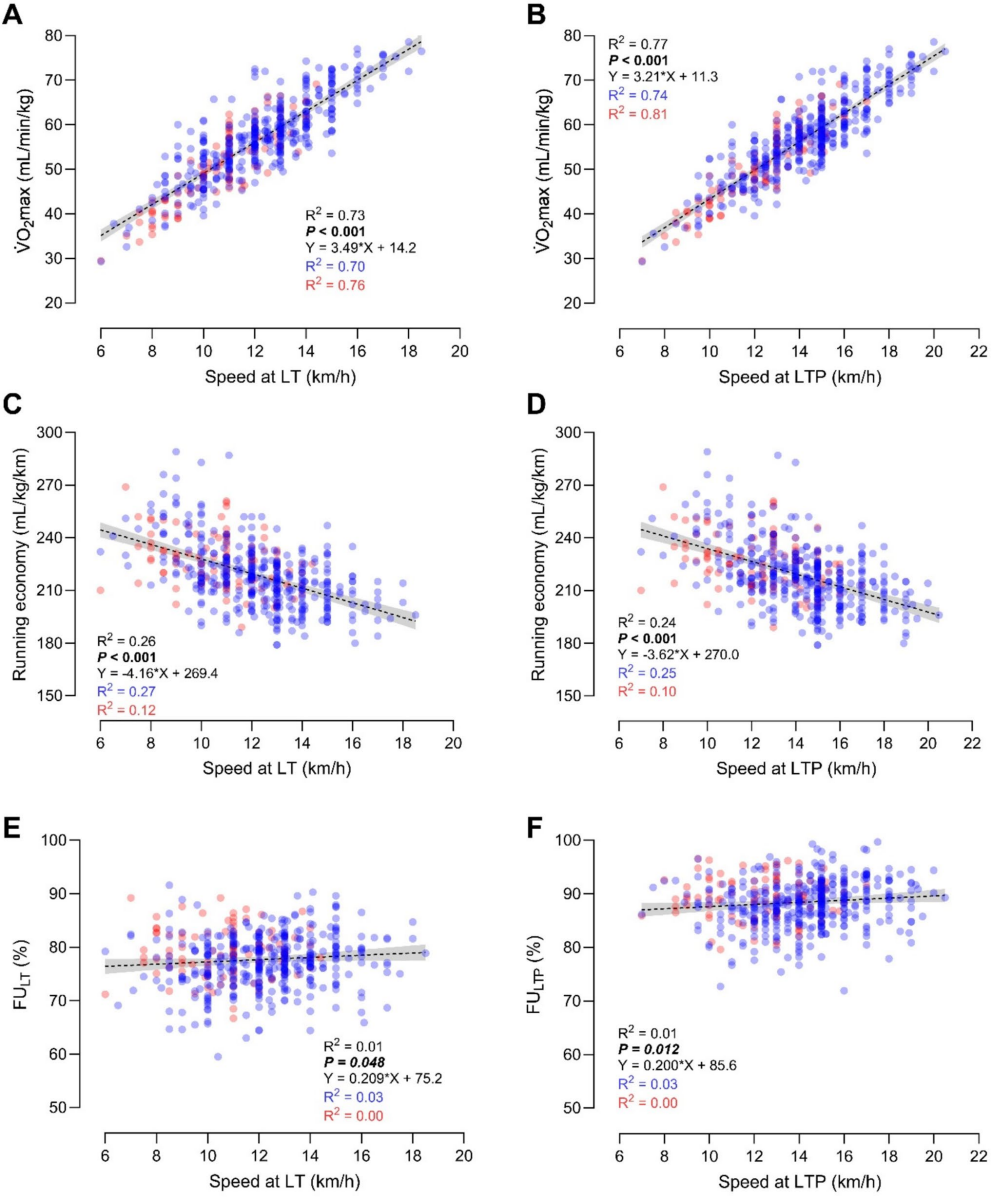
Correlations between speed at lactate threshold (LT) or lactate turnpoint (LTP) and maximal oxygen uptake (${\dot{\mathrm{V}}}{\mathrm{O}}_2 {\mathrm{max}}$) (**A**, **B)**, running economy (**C**, **D**), and fractional utilisation at lactate threshold (FU_LT_) and fractional utilisation at lactate turnpoint (FU_LTP_) (**E**, **F)** in runners. The *dotted line* represents the linear regression (and 95% confidence intervals in *grey shading*), *blue dots* represent male athletes (*n* = 390), and *red dots* represent female athletes (*n* = 105)

**Fig. 3:**
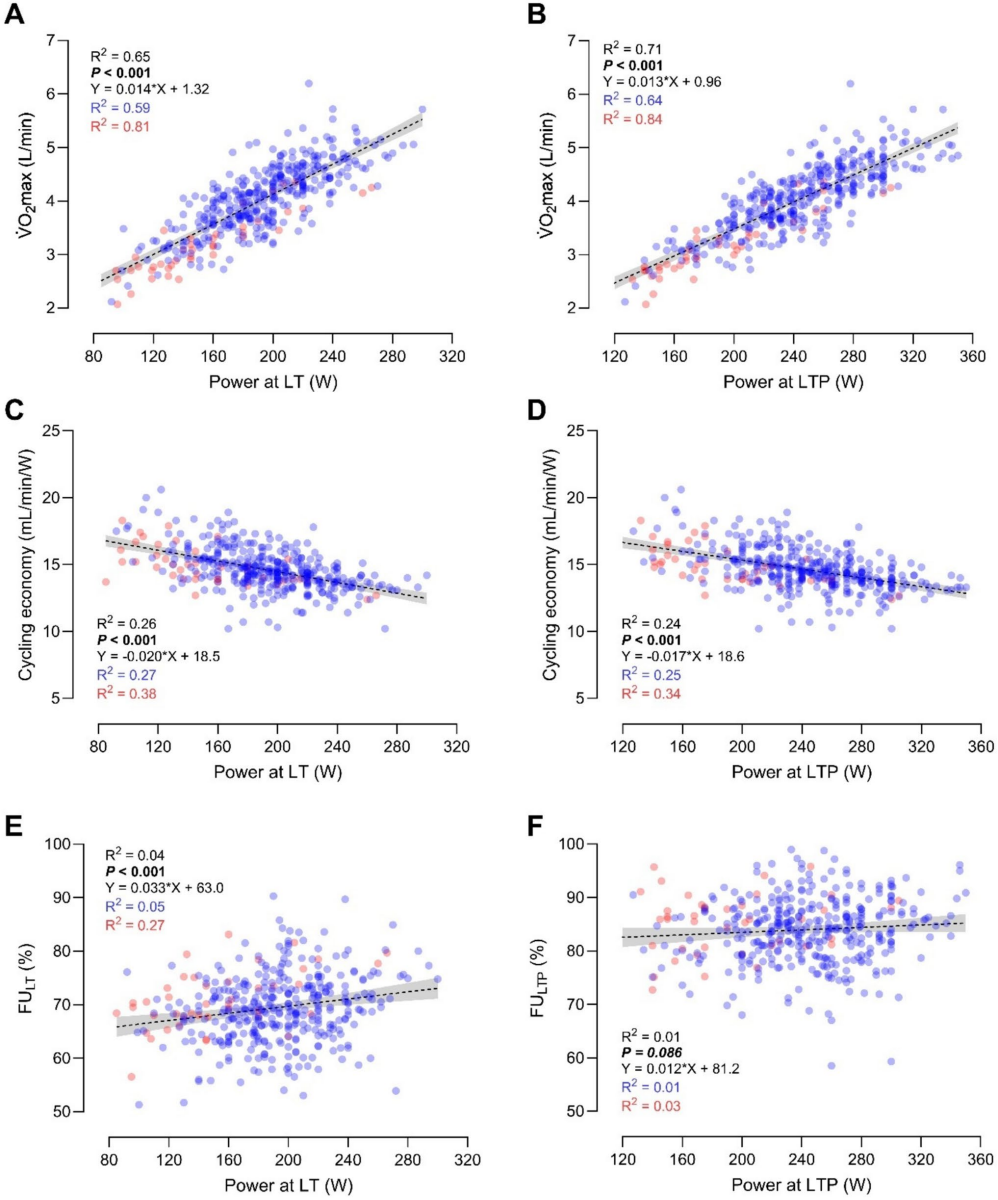
Correlations between absolute power output at lactate threshold (LT) or lactate turnpoint (LTP) and maximal oxygen uptake (${\dot{\mathrm{V}}}{\mathrm{O}}_2 {\mathrm{max}}$) (**A**, **B)**, cycling economy (**C**, **D**), and fractional utilisation at lactate threshold (FU_LT_) and fractional utilisation at lactate turnpoint (FU_LTP_) (**E**, **F**) in cyclists. The *dotted line* represents the linear regression (and 95% confidence intervals with *grey shading*), *blue dots* represent males (*n* = 351), and *red dots* represent females (*n* = 42)

The correlations between ${\dot{\mathrm{V}}}{\mathrm{O}}_2 {\mathrm{max}}$, RE or CE, FU_LT_, and FU_LTP_ and speed or power at LT and LTP (i.e. performance proxies) for all participants are illustrated in Fig. 2 for running, and in Fig. 3 for cycling. In running, speed at LT and LTP were positively correlated with ${\dot{\mathrm{V}}}{\mathrm{O}}_2 {\mathrm{max}}$ (*R*^2^ ≥ 0.73; *P* < 0.001 for both sexes) and negatively correlated with RE (*R*^2^ ≥ 0.25; *P* < 0.001 for both sexes; indicating a better RE for faster runners), while no correlations were found with FU_LT_ (*R*^2^ = 0.009) and FU_LTP_ (*R*^2^ = 0.013). In cycling, power at LT and LTP were also positively correlated with ${\dot{\mathrm{V}}}{\mathrm{O}}_2 {\mathrm{max}}$ (*R*^2^ ≥ 0.65; *P* < 0.001 for both sexes) and FU_LT_ in females (*R*^2^ = 0.27; *P* < 0.001), and negatively correlated with RE (*R*^2^ ≥ 0.24; *P* < 0.001 for both sexes), with no correlations with FU_LTP_ (*R*^2^ = 0.007) or FU_LT_ in males (*R*^2^ ≥ 0.047; *P* < 0.001).

**Fig. 4:**
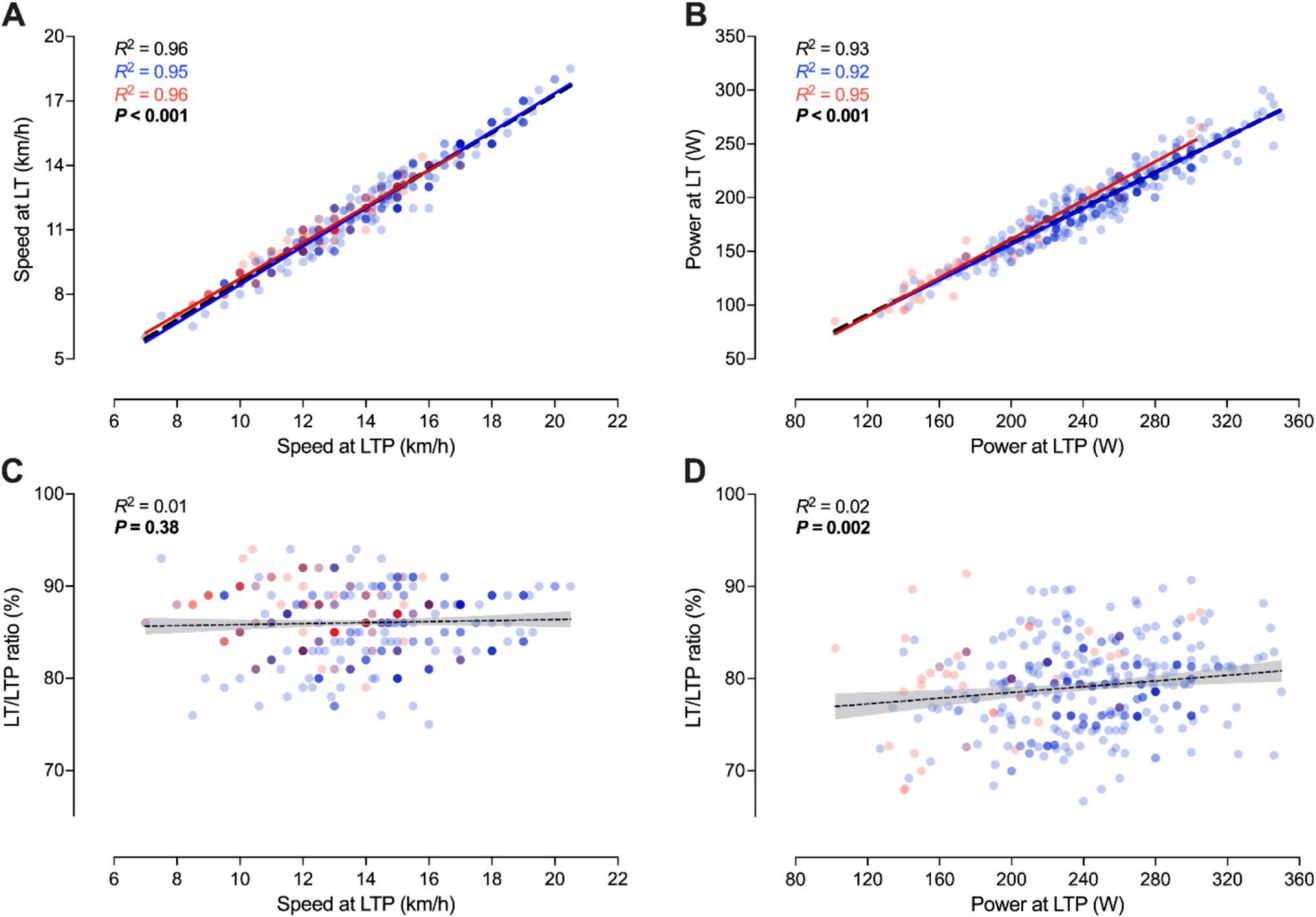
Correlations between speed or power at lactate threshold (LT) and lactate turnpoint (LTP) (**A**, **B**), and between the LT/LTP ratio and speed or power at LTP (**C**, **D**). The *dashed black line* corresponds to regressions for all participants, whilst the *blue and red lines* correspond to males and females, respectively. In panels **C** and **D**, the *grey shading* indicates the 95% confidence intervals (*n* = 495 for running; *n* = 393 for cycling)

A positive correlation between speed or power at LT and speed or power at LTP was found in both sexes and exercise modalities (*R*^2^ ≥ 0.93; all *P* < 0.001). The LT/LTP ratios were not correlated to speed or power at LTP in running (*R*^2^ = 0.001; *P* = 0.38) or cycling (*R*^2^ = 0.025; *P* = 0.002), indicating that LT/LTP ratios may be similar between athletes of different performance levels (Fig. 4). The LT/LTP ratio also showed narrow and consistent 95% confidence intervals across sexes in both running (all: 86%; males: 86–87%; females: 86%) and cycling (all: 79–80%; males: 79–80%; females: 78–81%).

**Fig. 5:**
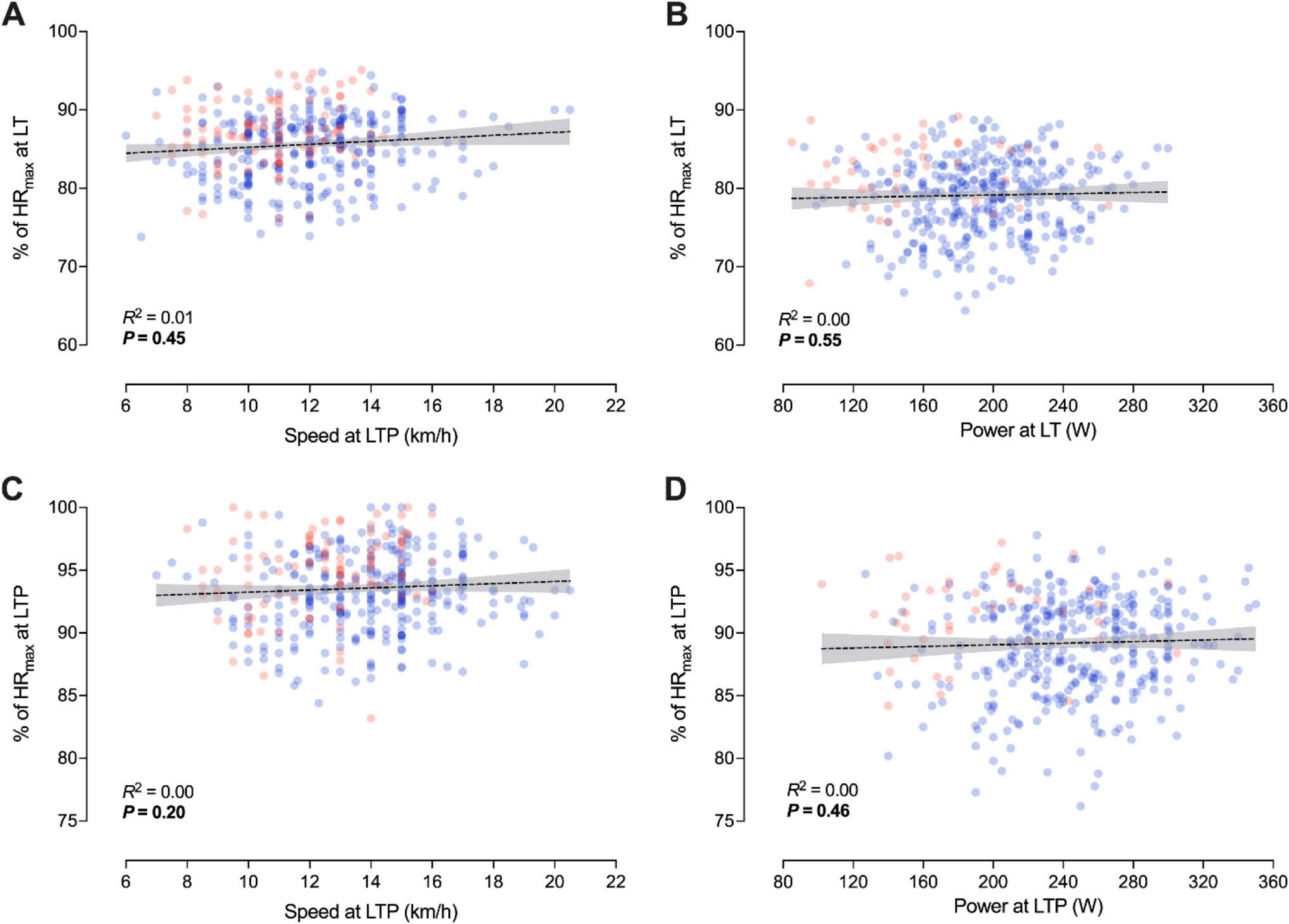
Correlations between speed or power at lactate threshold (LT) and % of maximum heart rate (HR_max_) at LT (**A**, **B**), and between speed or power at lactate turnpoint (LTP) and % of HR_max_ at LTP (**C**, **D**). The *dashed black line* corresponds to regressions for all participants, whilst the *blue and red lines* correspond to males and females, respectively. The *light grey areas* indicate the 95% confidence intervals (*n* = 495 for running; *n* = 393 for cycling)

No correlations were found between speed or power at LT and % of HR_max_ at LT or for the same variables corresponding to LTP (Fig. 5). The % of HR_max_ at LT or LTP showed narrow and consistent 95% confidence intervals across sexes in both running (LT: 85–86%; LTP: 93–94%) and cycling (LT: 79–80%; LTP: 89–90%).

### Performance Tertile Differences

In running, ${\dot{\mathrm{V}}}{\mathrm{O}}_2 {\mathrm{max}}$ increased across performance tertiles (all *P* < 0.001), whilst RE was better in highest versus lowest tertile (*P* = 0.004) but not between other tertiles (*P* = 0.051 middle vs highest tertile; *P* = 0.327 lowest vs middle tertile; Table 2) in females. No effects were detected for FU_LT_, FU_LTP_ and LT/LTP ratio (*P* ≥ 0.45; Table 2). In males, ${\dot{\mathrm{V}}}{\mathrm{O}}_2 {\mathrm{max}}$, RE and LT/LTP ratio were better in highest vs middle tertile, and in middle vs lowest tertile (all *P* < 0.001; Table 2). FU_LT_ and FU_LTP_ were higher in highest versus lowest tertile (*P* ≤ 0.001) but not between other tertiles (*P* ≥ 0.073; Table 2).

**Table 2:** Differences in participant characteristics across performance tertiles for the running tests

	Males (*n* = 390)				Females (*n* = 105)			
	Lowest tertile *n* = 131	Middle tertile *n* = 139	Highest tertile *n* = 120	Main effect	Lowest tertile *n* = 36	Middle tertile *n* = 33	Highest tertile *n* = 36	Main effect
Speed at LT, km/h	9.9 (1.1) [6.0; 11.4]	12.4 (0.5) [11.5; 13.0]^b^	14.7 (1.2) [13.1; 18.5]^b,c^	η^2^_p_ = 0.80 *P* < 0.001 F = 795.3	8.7 (1.0) [6.0; 10.0]	10.9 (0.3) [10.2; 11.6]^b^	12.8 (0.7) [11.8; 14.4]^b,c^	η^2^_p_ = 0.84 *P* < 0.001 F = 264.2
Speed at LTP, km/h	11.8 (1.4) [7.0; 14.5]	14.5 (0.7) [12.5; 16.0]^b^	16.9 (1.4) [14.5; 20.5]^b,c^	η^2^_p_ = 0.75 *P* < 0.001 F = 582.8	10.0 (1.2) [7.0; 12.0]	12.7 (0.5) [11.5; 14.0]^b^	14.7 (0.8) [13.0; 17.0]^b,c^	η^2^_p_ = 0.84 *P* < 0.001 F = 251.4
${\dot{\mathrm{V}}}{\mathrm{O}}_2 {\mathrm{max}}$, mL/min/kg	49.4 (6.3) [29.3; 65.7]	57.6 (4.8) [45.3; 72.5]^b^	65.1 (6.5) [49.5; 80.7]^b,c^	η^2^_p_ = 0.54 *P* < 0.001 F = 224.0	42.2 (5.1) [29.5; 52.0]	52.6 (5.0) [44.9; 62.3] ^#^	59.1 (5.1) [49.2; 69.1]^b,c^	η^2^_p_ = 0.67 *P* < 0.001 F = 99.2
RE, mL/kg/km	229 (20) [190; 289]	216 (14) [179; 252]^b^	209 (13) [180; 247]^b,c^	η^2^_p_ = 0.21 *P* < 0.001 F = 52.2	232 (18) [202; 292]	228 (17) [189; 261]	220 (12) [200; 246]^b^	η^2^_p_ = 0.10 *P* = 0.004 F = 5.8
FU_LT_,%	76 (5) [59; 92]	77 (5) [63; 86]	79 (5) [64; 90]^b^	η^2^_p_ = 0.01 *P* = 0.002 F = 6.3	79 (5) [71; 89]	79 (6) [67; 89]	79 (4) [72; 87]	η^2^_p_ = 0.00 *P* = 0.935 F = 0.1
FU_LTP_, %	87 (4) [73; 97]	88 (4) [77; 99]	89 (4) [72; 99]^b^	η^2^_p_ = 0.04 *P* < 0.001 F = 7.4	89 (3) [80; 97]	89 (4) [80; 96]	90 (4) [82; 96]	η^2^_p_ = 0.00 *P* = 0.826 F = 0.2
LT/LTP ratio, %	84 (4) [71; 94]	86 (3) [75; 94]^b,c^	87 (2) [82; 93]^b,c^	η^2^_p_ = 0.12 *P* < 0.001 F = 27.0	87 (3) [81; 94]	86 (3) [79; 92]	87 (2) [82; 92]	η^2^_p_ = 0.02 *P* = 0.452 F = 0.8

Mean (standard deviation) [minimum; maximum]

*FU*_*LT*_ fractional utilisation at lactate threshold, *FU*_*LTP*_ fractional utilisation at lactate turnpoint, *LT* lactate threshold, *LTP* lactate turnpoint, *RE* running economy, $\dot{{V}}{{O}}_2 {{max}}$ maximal oxygen uptake

^a^Sex differences independently of tertile

^b^Different from lowest tertile

^c^Different from middle tertile

In cycling, ${\dot{\mathrm{V}}}{\mathrm{O}}_2 {\mathrm{max}}$ increased across performance tertiles (all *P* < 0.001) and cycling economy was worse in lowest compared with middle tertile (*P* = 0.003) and highest tertile (*P* < 0.001) but did not differ between middle and highest tertiles (*P* = 0.084; Table 3), in females. Similarly, FU_LT_ and LT/LTP ratio were lower in lowest compared to middle tertile (*P* ≤ 0.010) and highest tertile (*P* ≤ 0.006), but not between middle and highest tertiles (*P* ≥ 0.190; Table 3). No tertile effects were detected for FU_LTP_. In males, ${\dot{\mathrm{V}}}{\mathrm{O}}_2 {\mathrm{max}}$, cycling economy and LT/LTP ratio improved across performance tertiles (*P* ≤ 0.011), while FU_LT_ was lower in lowest compared with middle tertile (*P* = 0.019) and highest tertile (*P* = 0.002), but not between middle and highest tertiles (*P* = 0.394; Table 3). No tertile effects were detected for FU_LTP_.

**Table 3:** Differences in participant characteristics across performance tertiles for the cycling tests

	Males (*n* = 351)				Females (*n* = 42)			
	Lowest tertile *n* = 116	Middle tertile *n* = 117	Highest tertile *n* = 118	Main effect	Lowest tertile *n* = 13	Middle tertile *n* = 15	Highest tertile *n* = 14	Main effect
Power at LT, W	154 (20) [92; 179]	193 (9) [180; 209]^b^	234 (20) [210; 300]^b,c^	η^2^_p_ = 0.79 *P* < 0.001 F = 656.2	108 (13) [85; 127]	145 (11) [130; 160]^b^	204 (30) [162; 266]^b,c^	η^2^_p_ = 0.80 *P* < 0.001 F = 79.7
Power at LTP, W	202 (26) [127; 250]	244 (16) [211; 280]^b^	289 (22) [250; 350]^b,c^	η^2^_p_ = 0.73 *P* < 0.001 F = 475.1	145 (18) [102; 175]	179 (18) [145; 205]^b^	246 (30) [210; 305]^b,c^	η^2^_p_ = 0.78 *P* < 0.001 F = 70.7
${\dot{\mathrm{V}}}{\mathrm{O}}_2 {\mathrm{max}}$, L/min	3.56 (0.47) [2.12; 4.91]	4.05 (0.43) [3.19; 5.09]^b^	4.61 (0.43) [3.44; 6.20]^b,c^	η^2^_p_ = 0.48 *P* < 0.001 F = 163.2	2.61 (0.43) [1.7; 3.45]	2.98 (0.24) [2.53; 3.30]^b^	3.83 (0.38) [3.32; 4.36]^b,c^	η^2^_p_ = 0.69 *P* < 0.001 F = 42.6
CE, mL/min/W	15.6 (1.7) [10.2; 20.6]	14.5 (1.2) [10.3; 17.3]^b^	13.9 (1.1) [10.2; 17.8]^b,c^	η^2^_p_ = 0.21 *P* < 0.001 F = 45.3	16.0 (1.3) [13.7; 18.3]	14.7 (1.2) [12.7; 17.1]^b^	13.9 (0.8) [12.4; 15.4]^b^	η^2^_p_ = 0.41 *P* < 0.001 F = 13.3
FU_LT_, %	68 (5) [51; 82]	70 (7) [54; 90]^b^	70 (6) [53; 90]^b^	η^2^_p_ = 0.04 *P* = 0.002 F = 6.5	67 (4) [57; 73]	71 (6) [63; 83]^b^	74 (5) [64; 82]^b^	η^2^_p_ = 0.22 *P* = 0.008 F = 5.5
FU_LTP_, %	83 (5) [68; 96]	84 (7) [58; 99]	84 (6) [59; 96]	η^2^_p_ = 0.00 *P* = 0.749 F = 0.3	84 (7) [73; 96]	85 (4) [75; 90]	86 (6) [77; 96]	η^2^_p_ = 0.02 *P* = 0.642 F = 0.4
LT/LTP ratio, %	76 (5) [58; 86]	79 (4) [69; 90]^b^	81 (4) [72; 91]^b,c^	η^2^_p_ = 0.16 *P* < 0.001 F = 33.3	75 (6) [64; 84]	81 (5) [72; 91]^b^	83 (4) [75; 87]^b^	η^2^_p_ = 0.30 *P* < 0.001 F = 8.5

Mean (standard deviation) [minimum; maximum]

*CE* cycling economy, *FU*_*LT*_ fractional utilisation at lactate threshold, *FU*_*LTP*_ fractional utilisation at lactate turnpoint, *LT* lactate threshold, *LTP* lactate turnpoint, $\dot{{V}}{{O}}_2 {{max}}$ maximal oxygen uptake

^a^Sex differences independently of tertile

^b^Different from lowest tertile

^c^Different from middle tertile

### Multiple Linear Regression Models

In running, the following equations were developed from multiple linear regression models from ${\dot{\mathrm{V}}}{\mathrm{O}}_2 {\mathrm{max}}$ (mL/kg/min), RE (mL/kg/km), and FU_LT_ and FU_LTP_ (%) to estimate speed at LT and LTP (km/h; Table 4):

1 $${\text{Speed at LT}} = {-} \, 0.365 + 0.213 \times {{\dot{\mathrm{V}}}\mathrm{O}}_{2} \max {-} 0.051 \times {\mathrm{RE}} + 0.149 \times {\mathrm{FU}}_{{{\mathrm{LT}}}} ,$$

2 $${\text{Speed at LTP}} = {-}\;0.619 + 0.235 \times {{\dot{\mathrm{V}}}\mathrm{O}}_{2} \max {-} 0.051 \times {\mathrm{RE}} + 0.142 \times {\mathrm{FU}}_{{{\mathrm{LTP}}}} .$$

**Table 4:** Equation coefficients and R^2^ of the multiple linear regression models to estimate speed/power at LT or LTP from physiological determinants

Exercise modality	Sex	Determinant	Speed or power at LT			Speed or power at LTP		
			*R*^*2*^	Coefficient	95% CI	*R*^*2*^	Coefficient	95% CI
Running	All	*Constant*	0.99	* − 0.37*	*(− 0.95, 0.23)*	0.97	* − 0.62*	*(− 1.63, 0.40)*
		${\dot{\mathrm{V}}}{\mathrm{O}}_2 {\mathrm{max}}$		0.21	(0.21, 0.22)		0.24	(0.23, 0.24)
		RE		* − *0.051	(*− *0.053, − 0.050)		* − *0.051	(*− *0.053, − 0.049)
		FU_LT_ or FU_LTP_		0.15	(0.15, 0.16)		0.14	(0.13, 0.15)
	Males	*Constant*	0.99	* − 0.36*	*(− 0.95, 0.23)*	0.97	* − 0.72*	*(− 1.81, 0.37)*
		${\dot{\mathrm{V}}}{\mathrm{O}}_2 {\mathrm{max}}$		0.21	(0.21, 0.22)		0.23	(0.23, 0.24)
		RE		* − *0.052	(*− *0.053, − 0.050)		* − *0.052	(*− *0.054, − 0.049)
		FU_LT_ or FU_LTP_		0.15	(0.15, 0.16)		0.15	(0.14, 0.16)
	Females	*Constant*	0.99	* − 0.34*	*(− 1.27, 0.58)*	0.96	* − 0.64*	*(− 3.07, 1.78)*
		${\dot{\mathrm{V}}}{\mathrm{O}}_2 {\mathrm{max}}$		0.21	(0.20, 0.21)		0.23	(0.22, 0.24)
		RE		* − *0.046	(*− *0.048, − 0.043)		* − *0.042	(*− *0.048, − 0.037)
		FU_LT_ or FU_LTP_		0.14	(0.13, 0.15)		0.12	(0.10 – 0.14)
Cycling	All	*Constant*	0.98	* − 4.67*	*(− 12.82, − 3.49)*	0.94	* − 15.31*	*(− 35.65, 5.03)*
		${\dot{\mathrm{V}}}{\mathrm{O}}_2 {\mathrm{max}}$		0.047	(0.046, 0.047)		0.056	(0.055, 0.058)
		CE		* − *11.98	(*− *12.32, − 11.64)		* − *12.22	(*− *12.96, − 11.50)
		FU_LT_ or FU_LTP_		2.67	(2.58, 2.75)		2.51	(2.32, 2.70)
	Males	*Constant*	0.98	* − 4.42*	*(− 12.73, 3.88)*	0.93	* − 12.13*	*(− 34.23, 9.97)*
		${\dot{\mathrm{V}}}{\mathrm{O}}_2 {\mathrm{max}}$		0.047	(0.046, 0.047)		0.055	(0.053, 0.057)
		CE		* − *12.22	(*− *12.56, − 11.87)		* − *12.36	(*− *13.14, − 11.58)
		FU_LT_ or FU_LTP_		2.71	(2.63, 2.80)		2.55	(2.35, 2.75)
	Females	*Constant*	0.99	* − 20.385*	*(− 53.26, –12.49)*	0.97	* − 14.74*	*(− 64.96, 35.49)*
		${\dot{\mathrm{V}}}{\mathrm{O}}_2 {\mathrm{max}}$		0.051	(0.048, 0.054)		0.063	(0.058, 0.067)
		CE		* − *9.72	(*− *11.01, − 8.43)		* − *10.50	(*− *12.35, − 8.64)
		FU_LT_ or FU_LTP_		2.23	(1.91, 2.55)		1.940	(1.49, 2.39)

*CE* cycling economy, *CI* confidence interval, *FU*_*LT*_ fractional utilisation at lactate threshold, *FU*_*LTP*_ fractional utilisation at lactate turnpoint, *LT* lactate threshold, *LTP* lactate turnpoint, *RE* running economy, $\dot{{V}}{{O}}_2 {{max}}$ maximal oxygen uptake

Similarly, in cycling, equations estimating power at LT and LTP (W) were developed from ${\dot{\mathrm{V}}}{\mathrm{O}}_2 {\mathrm{max}}$ (L/min), CE (mL/min/W) and FU_LT_/FU_LTP_ (%; Table 4):

3 $${\text{Power at LT}} = {-}\;4.667 + 0.047 \times {{\dot{\mathrm{V}}}\mathrm{O}}_{2} \max {-} 11.978 \times {\mathrm{CE}} + 2.665 \times {\mathrm{FU}}_{{{\mathrm{LT}}}} ,$$

4 $${\text{Power at LTP}} = {-}\;15.312 + 0.056 \times {{\dot{\mathrm{V}}}\mathrm{O}}_{2} \max {-} 12.223 \times {\mathrm{CE}} + 2.508 \times {\mathrm{FU}}_{{{\mathrm{LTP}}}} .$$

**Fig. 6:**
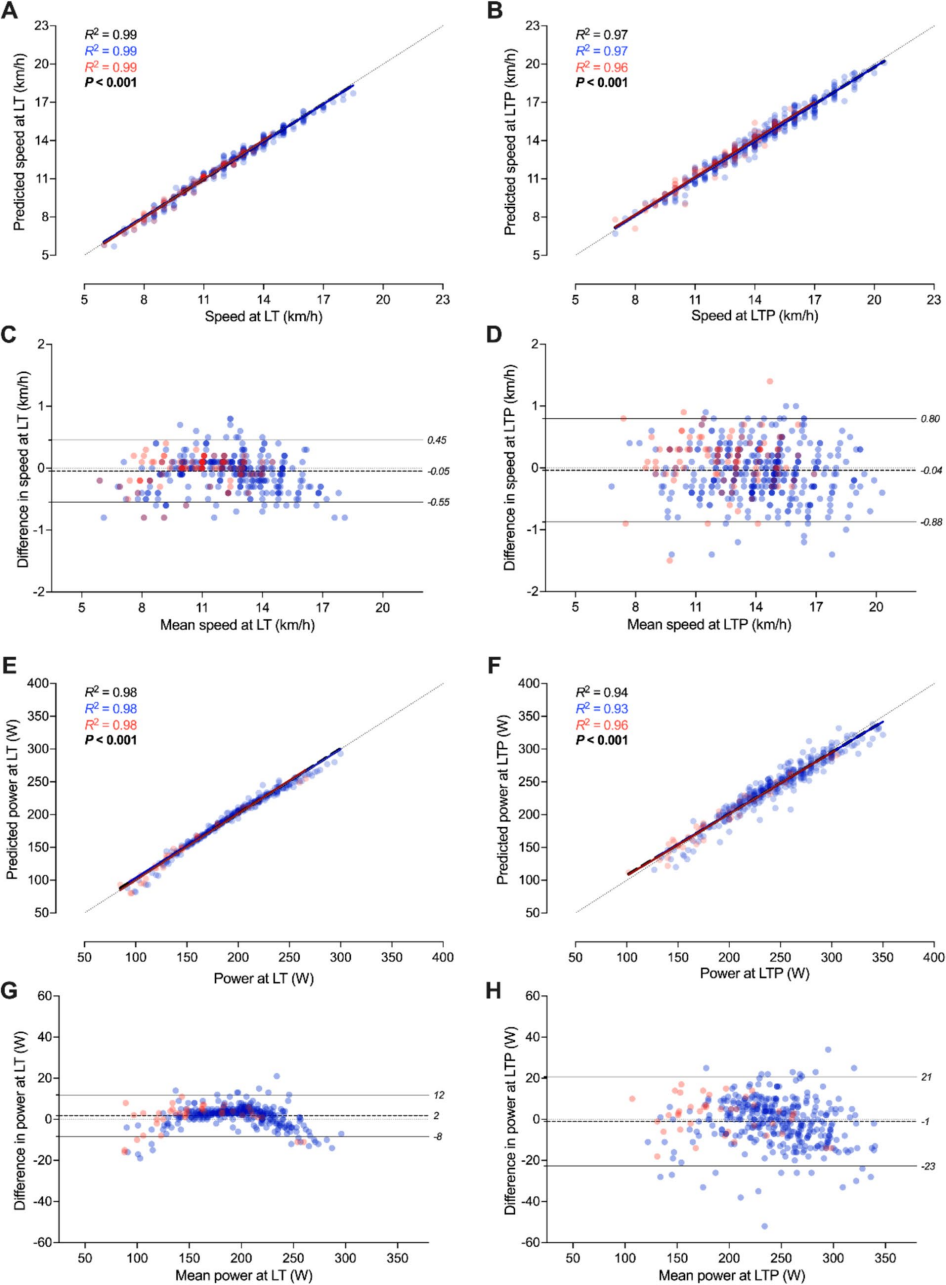
Comparisons between speed or power at lactate threshold (LT) and lactate turnpoint (LTP) and their prediction based on the coefficients of multiple linear regression analysis from Table 4. The *dotted line* corresponds to the reference line, whilst the *black*, *blue*, and *red lines* correspond the regressions for all participants, males, and females, respectively. The Bland–Altman plots display the differences between model predictions and speed or power at LT and LTP (*y-axis*) and their means (*x-axis*), with the *black dotted line* indicating bias and the *grey dotted lines* indicating the 95% limits of agreement (*n* = 495 for running; *n* = 393 for cycling)

For each model, the equation coefficients and R^2^ for all participants, males, and females are summarised in Table 4. ${\dot{\mathrm{V}}}{\mathrm{O}}_2 {\mathrm{max}}$, RE or CE, and FU_LT_ or FU_LTP_ significantly contributed to predicting performance proxies in all four models (all *P* < 0.001). The relationship between speed and power at LT or LTP and their predictions based on the above equations are reported in Fig. 6. Across all models, ten-fold cross-validation demonstrated very high predictive accuracy, with cross-validated *R*^*2*^ values that ranged from 0.94 to 0.99. Prediction errors ranged between 0.3 and 0.4 km/h for running and between 5 and 11 W for cycling.

### Relative Contribution of Performance Determinants

**Fig. 7:**
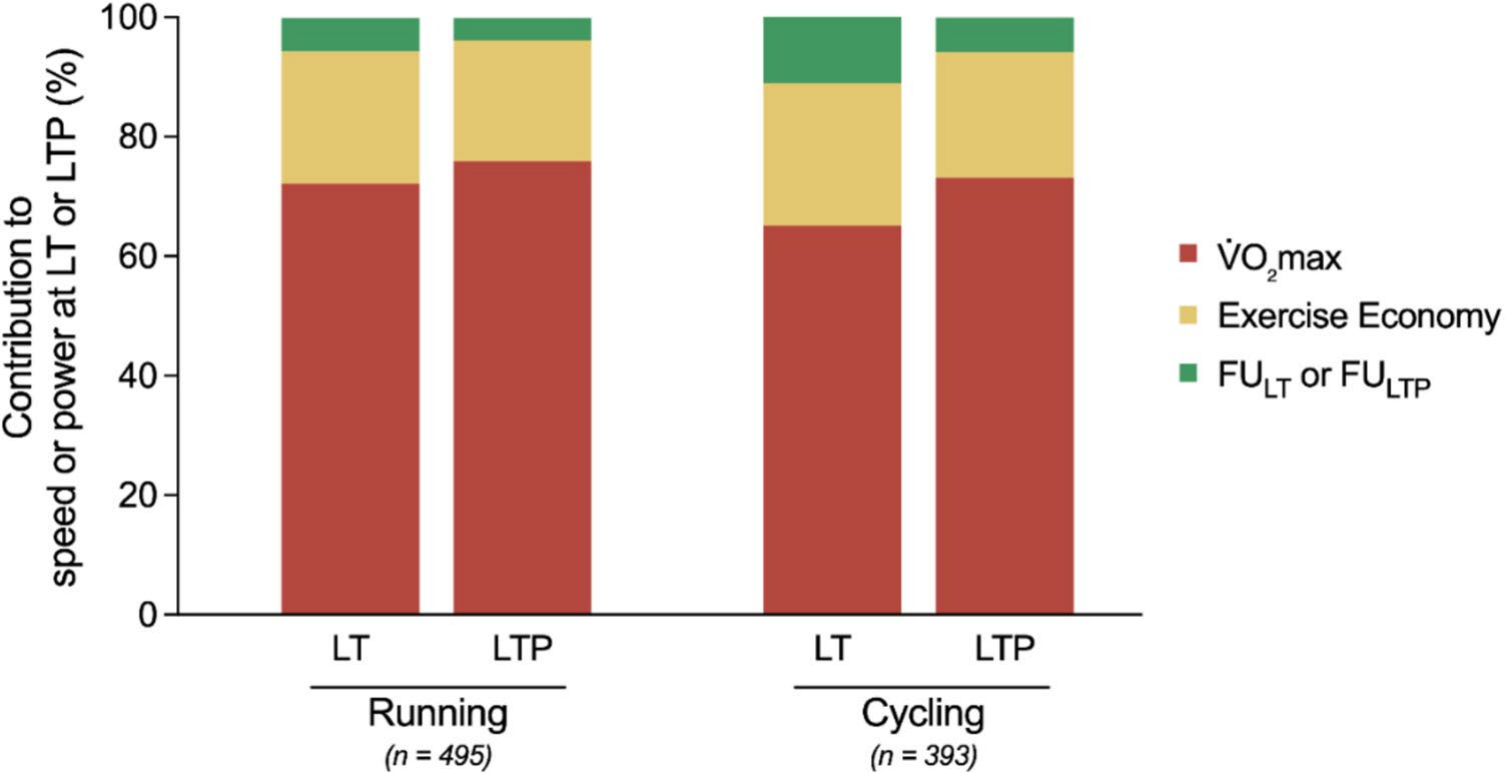
Relative contribution to speed or power at lactate threshold (LT) and lactate turnpoint (LTP) from maximal oxygen uptake (${\dot{\mathrm{V}}}{\mathrm{O}}_2 {\mathrm{max}}$), exercise economy (i.e. running or cycling economy), and fractional utilisation at lactate threshold (FU_LT_) and fractional utilisation at lactate turnpoint (FU_LTP_)

The relative contributions of ${\dot{\mathrm{V}}}{\mathrm{O}}_2 {\mathrm{max}}$, RE or CE, and FU_LT_ and FU_LTP_ to performance proxies are summarised in Table 5. For running, ${\dot{\mathrm{V}}}{\mathrm{O}}_2 {\mathrm{max}}$ was the primary determinant of both speed at LT (72%) and LTP (76%), with a slightly greater contribution in females compared with males (Table 5). Running economy contributed 22 and 20% to speed at LT and at LTP, respectively, whereas FU_LT_ and FU_LTP_ had a much smaller contribution (4–6%; Table 5, Fig. 7). In cycling, ${\dot{\mathrm{V}}}{\mathrm{O}}_2 {\mathrm{max}}$ was also found to be most influential, accounting for 65% and 73% of power at LT and LTP, respectively. Cycling economy contributed to 21–24%, while FU_LT_ and FU_LTP_ had an influence of only 6–11% (Table 5, Fig. 7).

**Table 5:** Relative contribution of ${\dot{\mathrm{V}}}{\mathrm{O}}_2 {\mathrm{max}}$, exercise economy, and FU_LT_ and FU_LTP_ to speed or power at LT and LTP for running and cycling, used as proxies of performance

Exercise modality	Performance proxy	Determinant	Contribution to performance proxies (%)		
			All	Males	Females
Running	Speed at LT (km/h)	${\dot{\mathrm{V}}}{\mathrm{O}}_2 {\mathrm{max}}$ (mL/min/kg)	72.2	70.0	80.7
		Economy (mL/kg/km)	22.1	22.3	14.8
		FU_LT_ (%)	5.6	7.3	4.5
	Speed at LTP (km/h)	${\dot{\mathrm{V}}}{\mathrm{O}}_2 {\mathrm{max}}$ (mL/min/kg)	75.9	73.6	85.9
		Economy (mL/kg/km)	20.2	20.9	11.7
		FU_LTP_ (%)	3.8	5.5	2.4
Cycling	Power at LT (W)	${\dot{\mathrm{V}}}{\mathrm{O}}_2 {\mathrm{max}}$ (L/min)	65.1	60.7	61.2
		Economy (mL/min/W)	23.9	26.3	22.3
		FU_LT_ (%)	11.0	12.9	16.4
	Power at LTP (W)	${\dot{\mathrm{V}}}{\mathrm{O}}_2 {\mathrm{max}}$ (L/min)	73.1	68.4	73.3
		Economy (mL/min/W)	21.1	24.1	22.2
		FU_LTP_ (%)	5.8	7.5	4.5

*CE* cycling economy, *FU*_*LT*_ fractional utilisation at lactate threshold, *FU*_*LTP*_ fractional utilisation at lactate turnpoint, *LT* lactate threshold, *LTP* lactate turnpoint, *RE* running economy, $\dot{{V}}{{O}}_2 {{max}}$ maximal oxygen uptake

## Discussion

This study examined the relationships between key physiological determinants of endurance performance (i.e. ${\dot{\mathrm{V}}}{\mathrm{O}}_2 {\mathrm{max}}$, exercise economy [RE/CE], and fractional utilisation of ${\dot{\mathrm{V}}}{\mathrm{O}}_2 {\mathrm{max}}$ [FU_LT_/FU_LTP_]) and performance proxies (i.e. speed or power at LT and LTP) in a large heterogeneous cohort of runners and cyclists from recreational to elite athletes. Our findings highlight that, in both exercise modalities, ${\dot{\mathrm{V}}}{\mathrm{O}}_2 {\mathrm{max}}$ is the primary determinant of endurance performance followed by exercise economy, which collectively account for 95% of performance variance. Conversely, fractional utilisation, although important in the determination of individual performance, only marginally explains between-athlete performance differences (< 5%).

### Maximal Oxygen Uptake (${\dot{\mathrm{V}}}{\mathrm{O}}_2 {\mathrm{max}}$)

In a heterogeneous cohort of runners and cyclists, ${\dot{\mathrm{V}}}{\mathrm{O}}_2 {\mathrm{max}}$ was the variable that correlated most strongly with performance (speed or power) at both LT and LTP (R^2^ ≥ 0.65). This was further supported by differences in ${\dot{\mathrm{V}}}{\mathrm{O}}_2 {\mathrm{max}}$ across performance tertiles, with athletes in the highest tertile displaying greatest ${\dot{\mathrm{V}}}{\mathrm{O}}_2 {\mathrm{max}}$ values. The present study reinforces the predominant role of ${\dot{\mathrm{V}}}{\mathrm{O}}_2 {\mathrm{max}}$ in performance prediction in both running [41–44], and cycling [5] and between the sexes.

Since the 1960s, a strong association between ${\dot{\mathrm{V}}}{\mathrm{O}}_2 {\mathrm{max}}$ and endurance running performance has been documented in populations with heterogeneous performance levels [41–44]. However, its predictive validity is reduced in runners with more homogeneous performance levels [45], indicating that additional physiological determinants contribute to performance. These include exercise economy and the fractional utilisation of ${\dot{\mathrm{V}}}{\mathrm{O}}_2 {\mathrm{max}}$ at a given running speed or cycling power. Costill and colleagues notably proposed that “marathon running success may, to a large part, be determined by running economy [i.e., exercise economy], max ${\dot{\mathrm{V}}}{\mathrm{O}}_2$ [i.e., ${\dot{\mathrm{V}}}{\mathrm{O}}_2 {\mathrm{max}}$] and the ability to utilize a large fraction of the max ${\dot{\mathrm{V}}}{\mathrm{O}}_2$ during marathon competition. [i.e., FU_LT_ or FU_LTP_]” [46] (p. 253). This informed Joyner’s model [1], which proposed that endurance performance is determined by the product of ${\dot{\mathrm{V}}}{\mathrm{O}}_2 {\mathrm{max}}$, exercise economy and the fractional utilisation of ${\dot{\mathrm{V}}}{\mathrm{O}}_2 {\mathrm{max}}$ at LT.

### Exercise Economy

Our results show that exercise economy also makes an important contribution to performance (> 20%) independently of ${\dot{\mathrm{V}}}{\mathrm{O}}_2 {\mathrm{max}}$, with both running and cycling economy demonstrating significant correlations with speed or power at LT and LTP (R^2^ ≥ 0.24). While the importance of RE is well established and widely acknowledged in the running literature [16, 47–49], the contribution of cycling economy (or efficiency) to endurance performance has historically been regarded as less influential and not different between populations of different performance levels [50–52]. Early physiological models often emphasised that cycling performance was determined primarily by ${\dot{\mathrm{V}}}{\mathrm{O}}_2 {\mathrm{max}}$ and LT, with gross efficiency playing a more limited role [54]. This is consistent with the greater mechanical constraint to movement in cycling, which would serve to reduce inter-individual variability, although inconsistency in methods of assessing efficiency may also play a role [55]. Nevertheless, studies have reported that cycling economy positively influences performance [56, 57], as supported in the present study. The stronger relationship observed in our study compared with previous studies [50–52] is also likely explained by the heterogeneous performance levels of the participants, allowing clearer distinctions in exercise economy between performance proxy categories.

### Fractional Utilisation of ${\dot{\mathrm{V}}}{\mathrm{O}}_2 {\mathrm{max}}$

In the present study, FU_LT_ and FU_LTP_ showed very limited or no association with performance proxies in runners or cyclists, indicating that they cannot be used to identify better-performing athletes in heterogeneous cohorts, as was recently reported by others [18, 20]. The small and inconsistent differences in FU_LT_ and FU_LTP_ across performance tertiles reinforces the limited discriminative value of FU in heterogeneous populations. In a heterogeneous cohort, ${\dot{\mathrm{V}}}{\mathrm{O}}_2 {\mathrm{max}}$ and exercise economy have the greatest influence on performance outcomes, thereby reducing the apparent contribution of FU_LT_ or FU_LTP_.

Importantly, FU_LT_ (and FU_LTP_) differs systematically between exercise modalities, with running typically eliciting higher values than cycling. Although submaximal running induces a higher ${\dot{\mathrm{V}}}{\mathrm{O}}_2$ than cycling at a matched external intensity, the involvement of the stretch–shortening cycle during running contrasts with the predominantly concentric nature of cycling, which is associated with a greater lactate accumulation and a larger glycolytic contribution to energy turnover, reflecting greater local muscular strain and altered muscle fibre recruitment patterns [58]. These mode-specific metabolic and neuromuscular constraints are strongly influenced by training background and movement familiarity [59–61], which may explain the lower fractional utilisation in cycling versus running.

Classic endurance models have long proposed that the ability to sustain a high fraction of ${\dot{\mathrm{V}}}{\mathrm{O}}_2 {\mathrm{max}}$ is a key determinant of endurance performance [1, 42, 54]. However, accumulating evidence indicates that fractional utilisation is a much more meaningful discriminator in highly trained or elite athletes, in whom ${\dot{\mathrm{V}}}{\mathrm{O}}_2 {\mathrm{max}}$ and economy values are tightly clustered and inter-individual variation is minimal [62].

### Ratio Between Performance Proxies

Performance at LT and LTP (R^2^ ≥ 0.93) were strongly correlated. The absence of correlations between LT/LTP ratios and performance proxies, together with the limited variability of LT/LTP ratios across the performance continuum and the narrow confidence intervals reported between sexes, suggests that speed or power at LT or LTP may be estimated with reasonable accuracy if either variable is known. These results could be used as a point of reference for applied practice, with a low or high LT/LTP ratio indicating potential for improvement through specific training for LT or LTP, respectively. It will be of interest for future research to assess the LT/LTP ratio longitudinally and in response to training interventions, which could provide further insights on the relationship between LT and LTP.

### Relative Contribution of Each Determinant

Regression models combining ${\dot{\mathrm{V}}}{\mathrm{O}}_2 {\mathrm{max}}$, economy and FU_LT_/FU_LTP_ explained the vast majority of variance in speed or power at LT (R^2^ ≥ 0.98) and LTP (R^2^ ≥ 0.94). This was expected, as speed or power at LT and LTP depend on the underlying physiological determinants. However, the very high agreement between the regression models and performance proxies, as well as the narrow limits of agreement of the Bland–Altman analysis, indicate that the relative contribution of each determinant is remarkably stable across participants of different characteristics and performance level. As a result, performance proxies can also be accurately estimated through the equations presented in the results. These results confirm that although ${\dot{\mathrm{V}}}{\mathrm{O}}_2 {\mathrm{max}}$ is the strongest predictor of performance (herein expressed as speed or power at LT and LTP), RE/CE make an additional important contribution, whilst fractional utilisation contributes to a smaller yet still meaningful extent. Furthermore, the stability of regression coefficients between sexes suggests that these prediction equations may be broadly applicable to all endurance-trained populations, although slight differences in RE/CE and ${\dot{\mathrm{V}}}{\mathrm{O}}_2 {\mathrm{max}}$ coefficients for females reflect expected physiological distinctions. Although sex differences in RE [14], ${\dot{\mathrm{V}}}{\mathrm{O}}_2 {\mathrm{max}}$ [22, 23], muscle size and fibre-type composition, cardiac and pulmonary dimensions, and haemoglobin mass [26, 27] have been reported, these differences do not seem to have a large influence on the relative contribution of physiological determinants to performance.

### Practical Applications

From a developmental perspective, ${\dot{\mathrm{V}}}{\mathrm{O}}_2 {\mathrm{max}}$ is mainly influenced by cumulative endurance training exposure [63, 64], although genetic factors and individual responses also contribute to its limits. While ${\dot{\mathrm{V}}}{\mathrm{O}}_2 {\mathrm{max}}$ remains trainable across the lifespan, periods of high physiological plasticity (typically during youth and early athletic development) appear to allow endurance training to elicit particularly robust central and peripheral adaptations, including cardiac remodelling, mitochondrial biogenesis and angiogenesis [65, 66]. Consequently, substantial endurance training exposure early in an athlete’s development may establish a high ${\dot{\mathrm{V}}}{\mathrm{O}}_2 {\mathrm{max}}$, after which further gains in ${\dot{\mathrm{V}}}{\mathrm{O}}_2 {\mathrm{max}}$ may be less consistent and with performance improvements being increasingly driven by other physiological determinants [4, 67].

The relevance of economy for endurance performance also has important training implications. In running, strength and plyometric training interventions have been shown to be among the most effective strategies to enhance RE in both a fresh and fatigued state [68, 69], and cross-sectional studies report that prolonged running bouts and high training volumes could also be beneficial for RE [35, 70, 71]. Improving cycling economy seems more challenging, and effective methods to enhance it are controversial [72]. Similar to running, interventions such as heavy strength training might offer positive adaptations through increased strength at the muscle fibre level, which could reduce muscle activation at a given absolute power/load [73, 74] and changes in muscle fibre composition [75, 76]. Longitudinal studies on elite endurance athletes also report improvements in exercise economy over multiple years of high training volumes [67, 77], indicating that this may be an effective stimulus for both runners and cyclists. Collectively, these data indicate that economy represents an important and modifiable contributor to endurance performance in both running and cycling in both males and females.

While ${\dot{\mathrm{V}}}{\mathrm{O}}_2 {\mathrm{max}}$ and economy are the strongest predictors of endurance performance in heterogeneous groups, the limited predictive value of FU_LT_ and FU_LTP_ does not imply that a high FU is undesirable or irrelevant for endurance athletes. Rather, it reflects that FU does not reliably differentiate performance in a heterogeneous population, and both high and low FU can be observed across the spectrum of endurance performance. Recreational athletes may display high FU simply because their ${\dot{\mathrm{V}}}{\mathrm{O}}_2 {\mathrm{max}}$ is low, meaning that their LT occurs at a relatively high fraction, while highly trained athletes may exhibit lower FU despite excellent performance because their ${\dot{\mathrm{V}}}{\mathrm{O}}_2 {\mathrm{max}}$ is substantially higher. Nonetheless, FU remains relevant for understanding an individual athlete’s physiological profile, for tracking longitudinal changes and for guiding race-specific strategies, particularly in elite athletes where performance differences are subtle and ${\dot{\mathrm{V}}}{\mathrm{O}}_2 {\mathrm{max}}$ is likely to already be maximised. Some excel through exceptionally high ${\dot{\mathrm{V}}}{\mathrm{O}}_2 {\mathrm{max}}$, others through superior exercise economy or fractional utilisation, suggesting that multiple physiological inputs may lead to comparable outcomes [3, 21]. For example, in our dataset, at a speed at LTP of 17 km/h, two runners displayed contrasting physiological profiles. One had a high ${\dot{\mathrm{V}}}{\mathrm{O}}_2 {\mathrm{max}}$ (74 vs 58 mL/kg/min), but worse RE (216 vs 192 mL/kg/km) and FU_LTP_ (82 vs 91%) while the other compensated for a lower ${\dot{\mathrm{V}}}{\mathrm{O}}_2 {\mathrm{max}}$ with superior economy and a higher FU_LTP._ Similarly, world-class runners such as Frank Shorter and Steve Prefontaine achieved similar personal best performances at 5 km (13:26.6 vs 13:21.9 min) and 10 km (27:45.9 vs 27:43.6 min) despite a large ${\dot{\mathrm{V}}}{\mathrm{O}}_2 {\mathrm{max}}$ difference (71.3 vs 84.4 mL/kg/min) [78], indicating that elite endurance performance can be underpinned by distinct physiological characteristics.

Although Joyner’s model is based on the above-described performance determinants, it has recently been recognised that their durability, or resilience, may also determine performance [9, 10]. Expanding the current findings to assess the contribution of physiological determinants to performance proxies in a state of acute fatigue (i.e. following prolonged exercise) could therefore be of interest. As the percentage of HR_max_ at LT and LTP, as defined in the present study (see Fig. 1), was found to be relatively consistent across individuals in both running (~ 86% and ~ 94%) and cycling (~ 80% and ~ 89%), submaximal HR targets could be used as a tool to guide training intensity for athletes without laboratory access, enabling them to estimate LT or LTP speeds or power outputs with reasonable accuracy in the field. However, regular assessments of ${\dot{\mathrm{V}}}{\mathrm{O}}_2 {\mathrm{max}}$, exercise economy, and speed or power at LTs provide coaches with a much more robust and precise approach for athlete profiling and monitoring and to guide training interventions.

## Methodological Considerations

Endurance performance was not directly assessed in this study. Instead, we relied on performance proxies (i.e. speed or power at LT and LTP) which, although widely used and strongly associated with performance, cannot fully reflect real competitive outcomes. Subsequently, the models should be interpreted as predicting physiological performance proxies rather than actual endurance performance. Direct measures such as race results would have provided a more complete picture of performance capacity for specific races, as speed or power at LT and LTP may not capture other factors influencing real-world outcomes (e.g. tactics, environment, or durability). Similarly, athletes’ training characteristics and main endurance event were not reported and could have had a small influence on our results. Second, the statistical power differed between sexes, owing to a substantially smaller female sample size. Therefore, some associations that were found to be significant in male athletes did not reach significance in female athletes despite similar effect sizes. Conversely, in male athletes, the very large sample size may have led to significant results that may not necessarily be meaningful in practical terms, especially when correlations involving single variables and performance tertile analysis elicited different results (e.g. FU_LT_ and FU_LTP_). Therefore, female-specific considerations should be interpreted with caution, especially in the cycling cohort. Third, CE was used as a performance determinant in the study, although gross efficiency has been used more broadly in cycling research. CE was used to reflect Joyner’s model of endurance performance and because it has been demonstrated that CE is closely associated with gross efficiency [55]. Moreover, economy and ${\dot{\mathrm{V}}}{\mathrm{O}}_2 {\mathrm{max}}$ were analysed in absolute terms in cycling and relative to body mass in running, which limits a direct comparison between exercise modalities. In this study, LT and LTP were defined using fixed increases in blood lactate concentrations (+ 0.4–0.5 and + 1.5 mmol/L), whereas alternative approaches may identify slightly different thresholds [79] and corresponding FU_LT_ and FU_LTP_ estimates. Finally, as this study was observational in nature, the reported relationships are associative and should not be interpreted with causal inference.

## Conclusions

In summary, the current study revealed that, across a large and heterogeneous cohort of runners and cyclists, and between the sexes, ${\dot{\mathrm{V}}}{\mathrm{O}}_2 {\mathrm{max}}$ was consistently the strongest predictor of performance proxies (i.e. speed or power at LT and LTP), reinforcing its long-established central role in endurance exercise physiology. Exercise economy also contributed meaningfully to performance, confirming previous findings in running, and highlighting the importance of economy to cycling performance, which has often been overlooked. In contrast, fractional utilisation of ${\dot{\mathrm{V}}}{\mathrm{O}}_2 {\mathrm{max}}$ showed limited discriminative power in heterogeneous cohorts to explain between-athlete performance differences, although it likely remains relevant in populations with homogeneous characteristics.

## Supplementary Information

Below is the link to the electronic supplementary material.

Supplementary file1 (DOCX 607 kb)

## Declarations

### Availability of Data and Material

Data are available on request from the corresponding author.
