# Supplementary material for: 35 Years of Joyner’s Endurance Performance Model: Assessing the Contribution of Physiological Determinants of Performance Proxies in 888 Individuals from Recreational to World Class
Source: Sports Med. 2026 May 3;56(8):2047–65. doi: 10.1007/s40279-026-02439-y (PMC13457331; doi:10.1007/s40279-026-02439-y)
Supplement: Supplementary file 1 [file 40279_2026_2439_MOESM1_ESM.docx]

***Supplementary materials***

**Title: 35 years of Joyner’s endurance performance model: Assessing the contribution of physiological determinants of performance proxies in 888 individuals ranging from recreational to world-class**

Running heading: 35 years of Joyner’s endurance model: physiological determinants in 888 athletes

**Authors:** Loïs Mougin^1 *#^, Stephen J. Bailey^1^, Andrew M. Jones^2^, Michael J. Joyner^3^, Stephen A. Mears^1^, Rhona Pearce^4^, Michele Zanini^1,5 *^

**Affiliations**

^1^School of Sport, Exercise and Health Sciences, Loughborough University, Loughborough, UK

^2^Department of Public Health and Sport Sciences, University of Exeter, Exeter, UK

^3^Department of Anesthesiology & Perioperative Medicine, Mayo Clinic, Rochester, Minnesota, USA

^4^Loughborough Sport, Loughborough University, Loughborough, UK

^5^School of Education, Childhood, Youth and Sport, The Open University, Milton Keynes, UK

* Shared first authorship

**# Correspondence**

Loïs Mougin (L.m.b.h.mougin@lboro.ac.uk); +44 1509 226391; School of Sport, Exercise and Health Sciences, Loughborough University, Loughborough, Leicestershire LE11 3TU, UK.

***Figure S1.*** Correlations between absolute power output at LT or LTP and V̇O_2_max (A, B). The dotted line represents the linear regression (and 95% confidence intervals), blue dots represent males (n=351), and red dots represent females (n=42).

***Table S1.*** Equation coefficients and R² of the multiple linear regression models to estimate speed or power at lactate threshold or lactate turnpoint from physiological determinants of endurance performance for each performance tertile.

|  |  | **Speed or Power at LT** | | | | **Speed or Power at LTP** | | | |
| --- | --- | --- | --- | --- | --- | --- | --- | --- | --- |
|  |  | **Coeff.** | **95% CI** | | ***R²*** | **Coeff.** | **95% CI** | | ***R²*** |
| Running  (Lowest tertile) | *C* | *0.41* | *-0.23* | *1.05* | 0.98 | *0.09* | *-1.70* | *1.85* | 0.92 |
|  | V̇O_2_max | 0.19 | 0.19 | 0.20 |  | 0.23 | 0.22 | 0.24 |  |
|  | RE | -0.040 | -0.042 | -0.039 |  | -0.042 | -0.045 | -0.038 |  |
|  | FU_LT_ - FU_LTP_ | 0.12 | 0.11 | 0.13 |  | 0.11 | 0.10 | 0.13 |  |
| Running  (Middle tertile) | *C* | *1.74* | *0.83* | *2.65* | 0.93 | *2.40* | *0.64* | *4.17* | 0.86 |
|  | V̇O_2_max | 0.19 | 0.18 | 0.20 |  | 0.21 | 0.19 | 0.22 |  |
|  | RE | -0.051 | -0.054 | -0.049 |  | -0.050 | -0.055 | -0.046 |  |
|  | FU_LT_ - FU_LTP_ | 0.14 | 0.13 | 0.15 |  | 0.13 | 0.11 | 0.14 |  |
| Running  (Highest tertile) | *C* | *-0.14* | *-1.00* | *0.71* | 0.98 | *-0.81* | *-2.68* | *1.07* | 0.93 |
|  | V̇O_2_max | 0.22 | 0.21 | 0.23 |  | 0.23 | 0.22 | 0.25 |  |
|  | RE | -0.06 | -0.06 | -0.58 |  | -0.060 | -0.065 | -0.055 |  |
|  | FU_LT_ - FU_LTP_ | 0.17 | 0.16 | 0.18 |  | 0.17 | 0.15 | 0.18 |  |
| Cycling  (Lowest tertile) | *C* | *3.41* | *-5.78* | *12.60* | 0.98 | *2.40* | *-24.82* | *29.62* | 0.92 |
|  | V̇O_2_max | 0.042 | 0.041 | 0.043 |  | 0.051 | 0.048 | 0.054 |  |
|  | CE | -9.18 | -9.55 | -8.81 |  | -8.74 | -9.66 | -7.82 |  |
|  | FU_LT_ - FU_LTP_ | 2.15 | 2.03 | 2.27 |  | 1.84 | 1.56 | 2.12 |  |
| Cycling  (Middle tertile) | *C* | *6.35* | *-0.51* | *13.20* | 0.98 | *21.15* | *-12.82* | *55.13* | 0.84 |
|  | V̇O_2_max | 0.046 | 0.045 | 0.047 |  | 0.054 | 0.050 | 0.058 |  |
|  | CE | -12.69 | -13.13 | -12.25 |  | -13.45 | -15.20 | -11.70 |  |
|  | FU_LT_ - FU_LTP_ | 2.63 | 2.54 | 2.71 |  | 2.40 | 2.03 | 2.73 |  |
| Cycling  (Highest tertile) | *C* | *-4.40* | *-13.73* | *4.73* | 0.97 | *-12.59* | *-52.58* | *27.39* | 0.84 |
|  | V̇O_2_max | 0.052 | 0.050 | 0.053 |  | 0.054 | 0.050 | 0.059 |  |
|  | CE | -16.80 | -17.29 | -16.32 |  | -14.00 | -15.80 | -12.21 |  |
|  | FU_LT_ - FU_LTP_ | 3.32 | 3.22 | 3.41 |  | 2.93 | 2.58 | 3.27 |  |

***Correlation Matrix***

***Figure S2.*** Correlation matrix of running performance proxies and physiological determinants.

***Figure S3.*** Correlation matrix of cycling performance proxies and physiological determinants
